# A Rare Case of Extra-Pulmonary Sarcoidosis With Only Initial Presentation of Hypercalcemia

**DOI:** 10.7759/cureus.45100

**Published:** 2023-09-12

**Authors:** Swapnil Surpur, Amandeep Singh, Jonathan Webb

**Affiliations:** 1 Internal Medicine, Jawaharlal Nehru Medical College, Belagavi, IND; 2 Nephrology, University of Kentucky, Lexington, USA

**Keywords:** atypical presentation of sarcoidosis, renal sarcoidosis, sarcoidosis hypercalcemia, calcitriol-mediated hypercalcemia, extra pulmonary manifestations of sarcoidosis

## Abstract

Sarcoidosis is a systemic disorder characterized by the aberrant development of granulomatous inflammation within various organs in the body. In over 90% of cases, sarcoidosis typically manifests initially in the intra-thoracic region, characterized by pulmonary involvement or mediastinal lymphadenopathy. It is rare for sarcoidosis to manifest exclusively as extra-thoracic involvement and even more rarely for hypercalcemia to be the only initial sign. We present a case of a 70-year-old female with hypercalcemia and elevated 1,25-dihydroxyvitamin D3 (1,25(OH)2 D) which raised the suspicion of a granulomatous disease. Granulomatous diseases increase levels of 1,25(OH)2 D via the abnormal expression of 1 alpha-hydroxylase enzyme; therefore, these conditions should be considered in the differential diagnosis when encountered with hypercalcemia. PET-CT showed increased FDG uptake in the reticuloendothelial system. An easily accessible inguinal lymph node biopsy was performed which revealed non-necrotizing granulomatous inflammation. Other causes of non-necrotizing granulomatous diseases, cancer, and lymphoma were ruled out, leading to sarcoidosis being considered as a possible diagnosis. When diagnosing sarcoidosis, other potential causes of granulomatous inflammation need to be ruled out definitively via laboratory findings, imaging, and tissue histopathology before initiation of treatment with steroids. Treatment with glucocorticoids remains the mainstay therapy of 1,25(OH)2 D-mediated hypercalcemia associated with sarcoidosis. The patient was accordingly treated with prednisone which led to the normalization of calcium and 1,25(OH)2 D levels within three weeks. Here, we discuss the clinical features and investigations of extra-pulmonary sarcoidosis for early diagnosis and management.

## Introduction

Sarcoidosis is a rare inflammatory condition leading to granuloma formation in various organs in the body but most commonly affects the pulmonary and reticuloendothelial systems. Pulmonary with concurrent extrapulmonary involvement occurs in 90% of the patients, whereas isolated extra-pulmonary sarcoidosis is seen only in 2% of the patients [1]. The most common extrapulmonary sites of primary sarcoidosis include the skin, eyes, lymph nodes, liver, heart, spleen, CNS, musculoskeletal system, kidneys, parotid, and other salivary glands. The systemic manifestations of extrapulmonary sarcoidosis vary depending on the organ involved. Occasionally, patients with extrapulmonary primary sarcoidosis may have no symptoms at all and be diagnosed incidentally during the evaluation of other disorders.

Extrapulmonary disease may manifest before, concurrent with, or after the development of pulmonary disease; thus, patients with extrapulmonary sarcoidosis are encountered with presentations depending on the location of the disease and the organ involved. The most common initial presentations of extrapulmonary involvement are skin lesions, arthralgia, and joint pains. Dysregulated vitamin D metabolism is another well‐recognized complication of sarcoidosis, resulting in hypercalcemia. Although hypercalcemia is a common manifestation of sarcoidosis, it is rarely the initial presentation [2]. Extrarenal synthesis of calcitriol (1,25(OH)2 D3) is central to the pathogenesis of abnormal calcium homeostasis in these cases. Immune cell 1α-hydroxylase expression is controlled predominantly by locally synthesized interferon-γ (IFN-γ), which is highly expressed in sites of granulomatous inflammation [3].

Diagnosis of sarcoidosis requires the exclusion of other granulomatous etiologies and is based on a suitable history and clinical findings. In patients with normal chest radiographs, assessment and monitoring of extrapulmonary sarcoidosis with other diagnostic modalities, including ultrasonography, chest CT, gallium scintigraphy, MRI, and PET-CT may be helpful [4]. The presence of non-necrotizing granulomas on histology is often conclusive of sarcoidosis in these cases. Extrapulmonary sarcoidosis is extremely rare and can, therefore, potentially be very difficult to diagnose. It is often misdiagnosed as other conditions due to vague symptoms and non-specific multisystemic presentations. This case highlights the importance of early intervention following aberration in serum calcium levels and other relevant biomarkers in order to halt the progression of extrapulmonary sarcoidosis and its associated complications.

## Case presentation

**Figure 1:**
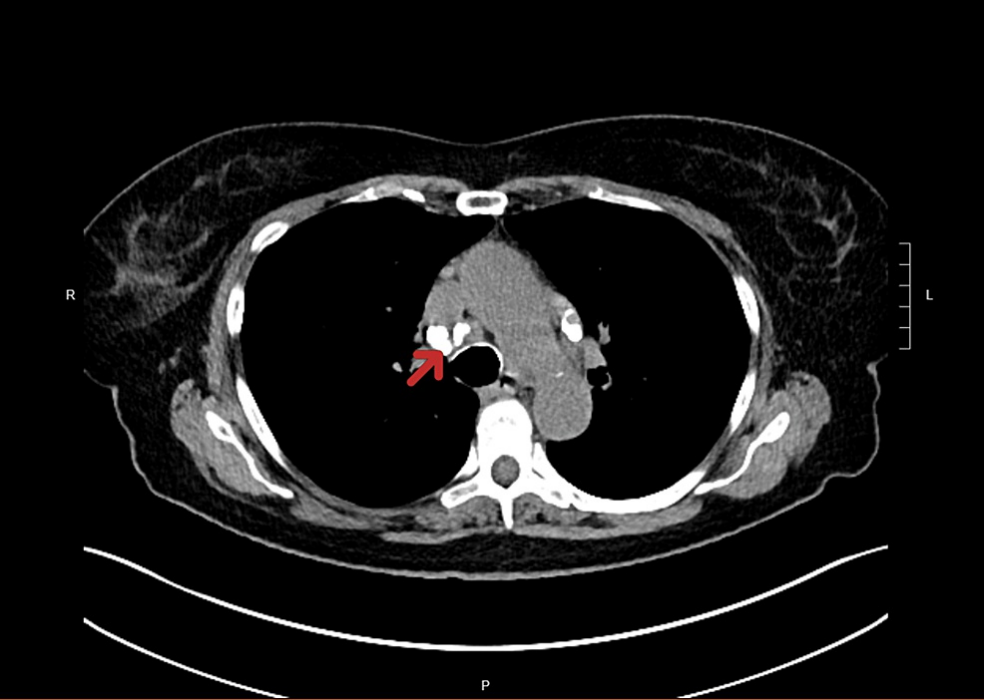
Calcification of the mediastinal lymph node

A 70-year-old Caucasian female with a history of childhood histoplasmosis was referred for the evaluation of hypercalcemia of 12.2 mg/dl and elevated 1,25-dihydroxyvitamin D3 (1,25(OH)2 D) of 123 pg/ml. She also gave the history of the increased number of kidney stones over the past two years with 10-15 in number during the last year alone. She also admitted to having complaints of increased constipation, muscle cramps in her legs, and on-and-off episodes of cramping type of abdominal pain during the same period. The patient had two past episodes of acute pancreatitis attributable to a presumptive diagnosis of sphincter of Oddi dysfunction. She admitted that she had not suffered any fractures during this period of the last two years but stated that she fractured her right lateral hand a few years ago secondary to trauma. Her chest CT showed a calcified lymph node in the mediastinum (Figure 1). The patient said that this finding initially appeared on her chest CT during her childhood when she had histoplasmosis at age 5. The same finding has been serially monitored and has remained unchanged on subsequent scans.

Her labs from the initial encounter were as follows: 1,25(OH)2 D levels were 123 pg/ml (normal reference in females: 18-78 pg/mL), 25-OH vitamin D was 29 ng/ml (normal reference: 30-50 ng/mL), serum calcium was 12.2 mg/dL (normal reference: 8.5-10.5 mg/dL), ionized calcium was 1.51 millimol/L (normal reference: 1.20 to 1.40 millimol/L), phosphorous was 3.6 mg/dL (normal reference: 3-4.5 mg/dL), and angiotensin-converting enzyme (ACE) levels were 165 U/L (normal reference: 16-85 U/L).

**Figure 2:**
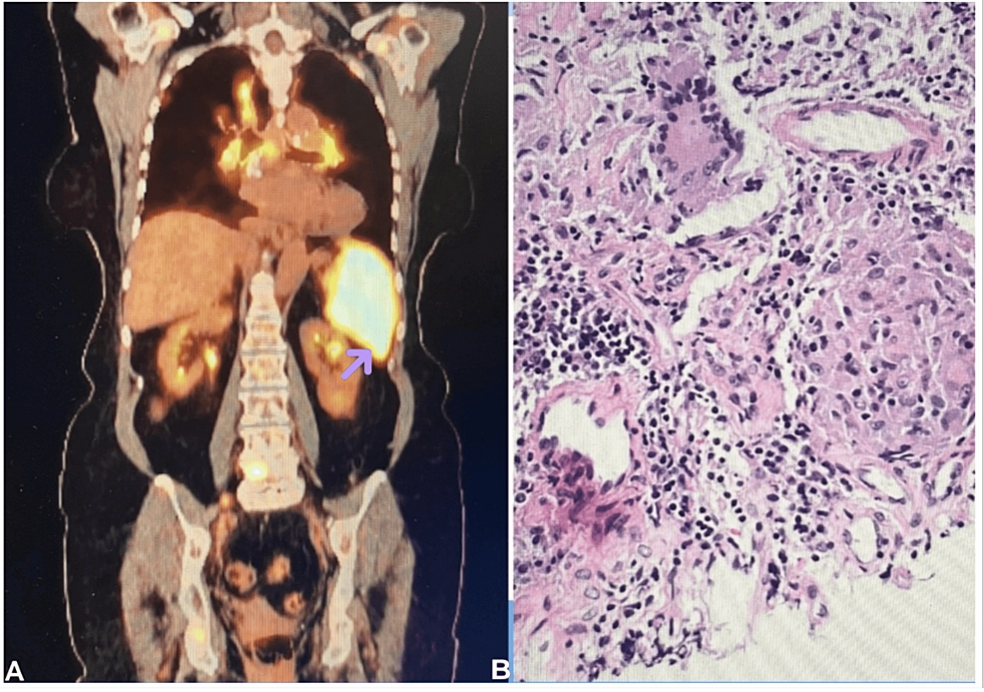
(A) FDG PET scan image shows extensive generalized lymphadenopathy with increased FDG uptake in the enlarged spleen. (B) Histological slide of the right inguinal lymph node biopsy shows the presence of non-necrotizing granulomatous inflammation FDG PET: F-18 fluorodeoxyglucose positron emission tomography

PET-CT showed extensive generalized lymphadenopathy with increased FDG uptake in the enlarged spleen (Figure 2A) as well as in the right inguinal lymph node which was biopsied due to its easy accessibility. The pathology of the biopsied lymph node revealed non-necrotizing granulomatous inflammation (Figure 2B). Staining with Grocott methenamine silver and results of Fungitell were negative for fungal organisms. AFB staining and QuantiFERON TB gold for the detection of *Mycobacterium tuberculosis* were also negative. Histopathology was negative for malignancy or lymphoma. Antibodies against histoplasmosis, blastomycosis, and coccidioidomycosis were all negative. Additionally, the myocardium showed no abnormal metabolic activity.

Treatment with prednisone at 0.5 mg per kg led to the normalization of calcium and 1,25(OH)2 D levels within three weeks. Her subsequent evaluation of serum calcium during her two-month follow-up showed levels of 10.1 mg/dL. ACE levels normalized to 52 U/L. Her symptoms gradually reduced in intensity, and her general condition is improving. The patient is currently being closely monitored through follow-up visits, which she continues to remain compliant with.

## Discussion

Granulomatous disease can lead to hypercalcemia and elevated 1,25(OH)2 D and, therefore, should be considered in the differential diagnosis of hypercalcemia. When diagnosing sarcoidosis, other potential causes of granulomatous inflammation are typically ruled out by clinical features, laboratory findings, imaging, and tissue histopathology. The diagnosis of sarcoidosis relies on three criteria: a compatible clinical and radiological presentation, biopsy evidence of non-necrotizing granulomas, and exclusion of other diseases with similar features [5].

A PET-CT scan is an essential tool in determining an appropriate biopsy site and excluding potential cardiac involvement of sarcoidosis. Sarcoidosis is an infiltrative disease that has a predilection to the interventricular septum, which due to its location can interfere with the normal functioning of the cardiac conduction system, leading to the development of cardiac blocks of varying degrees or tachyarrhythmias leading to sudden death [6]. Hence, in our case, we ruled cardiac involvement of sarcoidosis as it is essential to always exclude cardiac involvement in patients suspected of having sarcoidosis as cardiac involvement can lead to sudden cardiac death.

1,25(OH)2 D-mediated hypercalcemia is highly responsive to glucocorticoid therapy. Normalization of calcium levels is expected within a week after treatment with prednisone, 0.5 mg/kg daily (or equivalent). A relatively quick tapering (four to six weeks) can be attempted with frequent monitoring [7]. Patients in whom corticosteroids are relatively contraindicated or who relapse may be treated with hydroxychloroquine, chloroquine, or methotrexate as these have been shown to be effective in controlling hypercalcemia by reducing granuloma burden. Biologic agents are generally reserved for use when the aforementioned treatments fail to induce a meaningful clinical response. Infliximab is the most commonly used biological agent with the most robust data [8].

Although there is no consensus on follow-up times, an assessment at least every three to six months in the first two years has been proposed and thereafter yearly for the next three to five years, after which no more follow-up is necessary unless recurrence or new symptoms occur. Imaging is also important for monitoring disease course [9]. Follow-up surveillance of recurrence of pulmonary or extrapulmonary disease is done similarly to the initial work-up of the patient. Investigations like hemograms, renal and liver function tests, electrolytes especially calcium, inflammatory markers, ACE levels, and urinalysis including calcium and protein excretion levels are performed. The evaluation also includes pulmonary function tests, chest and abdominal radiography, complete eye examination, comprehensive bronchoalveolar lavage evaluation, cardiac examination with EKG, Mantoux testing with interferon-gamma release assay to exclude tuberculosis, and bone density scan for those treated with long-term corticosteroids. New organ involvement is reported to occur in nearly 25% of sarcoidosis patients at a two-year follow-up. Close monitoring of certain organ systems is particularly very important, as irreversible eye, cardiac, and neurological lesions may develop rapidly [5]. Therefore, our case highlights the need for further research on effective screening modalities to track the progression of extrapulmonary sarcoidosis and the need to develop sensitive biomarkers to diagnose and monitor the prognosis of the disease.

## Conclusions

Although sarcoidosis is a primarily intrathoracic condition, it can rarely present as an exclusively extra-thoracic disease process. Increased reporting of such cases improves understanding of the disease. Discussing such cases regularly can help physicians consider the possibility of the disease when nonspecific signs such as hypercalcemia occur, and there are no supporting symptoms before evaluating patients. There is also a lack of clinical studies on the management of extrapulmonary and extra-thoracic sarcoidosis. Hopefully, more effective markers will soon be found for the diagnosis and monitoring of the progression of extrapulmonary disease as some complications may set in rapidly and become challenging to treat.
